# When Fear Surrounding Childbirth Leads Women to Request a Planned Cesarean Birth

**DOI:** 10.1177/01939459211010192

**Published:** 2021-04-22

**Authors:** Janet Bryanton, Cheryl Tatano Beck, Stephanie Morrison

**Affiliations:** 1 Faculty of Nursing, University of Prince Edward Island, Charlottetown, PE, Canada; 2 School of Nursing, University of Connecticut, Storrs, CT, USA

**Keywords:** Fear surrounding childbirth, Planned cesarean birth, Maternal request for cesarean, Lived experience, Phenomenology, Qualitative

## Abstract

Fear surrounding childbirth requires a more in-depth understanding from women’s perspectives, especially those who request a planned cesarean due to that fear. Therefore, we explored primiparous and multiparous women’s lived experiences of fear surrounding childbirth in relation to their decision to request a planned cesarean birth. We used Colaizzi’s (1978) phenomenological method to interview 16 women from 4 provinces and to analyze the data. Women expressed numerous fears and most experienced more than one fear. Most feared their baby/babies being injured or dying during childbirth or developing complications themselves. Others feared experiencing a traumatic birth. Women described numerous emotional and physical manifestations of fear, and all believed that a planned cesarean birth would provide more control over the birth process. For some, the birth of their healthy baby/babies began a healing process, whereas others noted that their fear subsided or resolved upon confirmation that they would have a planned cesarean.

Although not a new phenomenon, fear surrounding childbirth is gaining increasing attention in clinical practice and research. Wijma (2003) suggested that fear of childbirth can occur on “a continuum, from almost no fear to extreme fear to give birth” (p. 141). In a recent systematic review, Nilsson et al. (2018) reported that 6.3–14.8% of women suffer from severe fear of childbirth and that rates vary by country. Fear can occur before pregnancy, prenatally, and during and following childbirth (Hildingsson et al., 2011). Dencker et al. (2019) reported similar levels of fear in primiparas and multiparas, although the reasons for fear varied between them. Childbirth fear affects women’s mental, physical, and psychosocial wellbeing. It is often accompanied by physical complaints, nightmares (Saisto et al., 2001), anxiety, eating disturbances (Nerum et al., 2006), depression (Humayun et al., 2013), and interference with occupational and academic functioning and with domestic and social activities and relationships (Wijma, 2003). Although there has been extensive quantitative research conducted regarding childbirth fears and their sources in general, there has been less focus on in-depth qualitative inquiry into the lived experience of women who suffer with fear surrounding childbirth, especially those who request a planned cesarean birth due to that fear.

## Understanding Fear Surrounding Childbirth

Numerous fears surrounding childbirth have been reported. These include fear of losing control, emergency cesarean birth (Eide et al., 2019), death or injury to themselves or the baby, inadequate support/care from care providers (Sheen & Slade, 2018), not having a voice in decision making, pain (Slade et al., 2019), epidural anesthesia (Poggi et al., 2018), episiotomies/perineal tears (Long et al., 2018), and the unknown (Sheen & Slade, 2018; Slade et al., 2019).

Women’s fears arise from many sources. A negative or traumatic experience with a previous birth, usually a difficult vaginal birth or an emergency cesarean, has been identified in numerous studies (Dencker et al., 2019; Eide et al., 2019; Karlström, 2017). Others include negative mood or depression (Størksen et al., 2015), negative stories told by others (Melender 2002), and previous perinatal loss (Melender, 2002). Additionally, exposure to information by the media (Stoll et al., 2014), previous infertility (Poikkeus et al., 2006), low self-efficacy or belief in one’s ability to give birth (Aval Nooghabi et al., 2019), and other traumatic life events such as childhood sexual abuse (Ryding et al., 2016) may be the underlying cause.

Although research regarding childbirth fear has been primarily quantitative, Wigert et al. (2020) performed a meta-synthesis of 14 good-quality studies, conducted in six countries, that used various qualitative methods related to a variety of aspects of women’s experiences of childbirth fear. The authors interpreted the experiences through the metaphor “being at a point of no return.” The three main themes identified were: “to suffer consequences from traumatic births,” “to lack warranty and understanding,” and “to face the fear.” Four studies included in the meta-synthesis, which were most closely related to the purpose or methodology of the current study, are described briefly. Nilsson and Lundgren (2009) found the essential theme of their study to be “losing oneself as a woman into loneliness.” Women described a loss of confidence in their ability to give birth, had feelings of failure turned inward, and felt weaker than and inferior to other women. They felt “captured” by the pregnancy and by the fact that they could not turn back. Nilsson et al. (2010) reported that previous childbirth experiences had a deep influence on women and were related to suffering and birth trauma. Women felt powerless after they tried to communicate with their midwives, who they described as “emotionless.” Health professionals were in charge and the focus was on techniques for a safe birth. Eriksson et al. (2006) found that women described their experience of fear in terms of “manifestations,” “judgements according to perceptions of self and others,” and “time and fluctuations.” They dealt with their fears by “evading,” “processing,” and “seeking help.” “Communicating fear” was not easy and it was influenced by “hindrances” such as their fear not being taken seriously and “preconditions” such as a midwife who showed understanding and legitimized their concerns. When Melender (2002) asked women how they dealt with their fears, four categories emerged: talking with others, writing about fears, obtaining knowledge, and reasoning that there was nothing to worry about.

Planned cesarean births have been associated with negative birth experiences and fear surrounding childbirth (Bryanton et al., 2008). They have also been associated with higher posttraumatic stress symptom levels in women (Beck et al., 2011). Researchers have reported that women across various cultures most commonly request a planned cesarean because of fear surrounding childbirth (Eide et al., 2019; Reyes & Rosenberg, 2019; Ryding et al., 2016). Reasons for this preference are similar to women in general who fear childbirth and include a previous negative birth experience (Ryding et al., 2016), a previous traumatic birth, fear of having an emergency cesarean, a deep-seated fear of childbirth since their early teens, fear of their own or their baby’s life being at risk, extreme pain, lack of control (Eide et al., 2019), depression, a history of abuse (Ryding et al., 2016), and episiotomy/perineal tears (Long et al., 2018). Long et al. (2018) conducted a qualitative meta-synthesis and uncovered four major themes regarding Chinese women’s preferences for cesarean birth: beliefs about cesarean birth; health care system factors; societal context and change; and women’s experiences of labor, vaginal birth, and cesarean. These themes reflected many of the aforementioned fears. Wijma (2003) suggested that women with severe childbirth fear are “forced to approach the. . . [birth], which is unknown, uncontrollable, and unavoidable” (p. 142), and the only option for them is to request an abortion or a cesarean birth.

The cesarean birth rate continues to increase internationally (Sandall et al., 2018); in Canada, the national cesarean birth rate in 2018–2019 was 29.4% (Canadian Institute Health Research [CIHI], 2020). No national statistics are available regarding maternal requests for planned cesareans. The Canadian health care system is universally funded; therefore, women do not pay for maternity care. Care providers include obstetricians, family physicians, midwives, nurse practitioners, nurses, and doulas; however, midwifery care is available in most but not all provinces. In 2018, the Society of Obstetricians and Gynecologists of Canada (SOGC) released its updated position statement related to maternal requests for cesarean birth. The SOGC recommended that reasons for maternal requests should be explored thoroughly over several sessions. “The discussion should highlight the person’s values, fears, or concerns central to their request” (p. 970). This may be done collaboratively with other members of the maternity care team or by referral to a perinatal mental health care expert. Evidenced-based risks and benefits should be discussed and a mutual decision on mode of birth should be made without coercion or bias (SOGC, 2018). Currently, there is no comprehensive system in each province to identify, refer for, and treat childbirth fear.

Assessment of fear and interventions to help reduce fear surrounding childbirth have been used in several countries such as Sweden for many years; however, Wulcan and Nilsson (2019) found that the approaches used by the midwives were not consistent. Stoll et al. (2018) purport that to develop strategies to address fear of childbirth requires an understanding of which interventions are most effective and the availability of validated assessment tools that are reflective of the comprehensive range of women’s fears surrounding childbirth. Based on a recent systematic review, few high-quality intervention studies have been conducted to investigate the effectiveness of various nonpharmacological interventions (Stoll et al., 2018). Of the seven studies included in this review, five were of moderate quality and two were strong. These studies were conducted in six countries, and the interventions varied from individual and group prenatal psychotherapy/education, intensive cognitive behavioral therapy, to prenatal Hatha yoga. Five of the seven studies reported significant reductions in fear due to the intervention; however, there was no consensus about the most effective intervention (Stoll et al., 2018).

Although numerous studies have been conducted regarding childbirth fears and their sources in general, there has been less in-depth inquiry into the lived experience of women who suffer with fear surrounding childbirth, especially those who request a planned cesarean birth. To our knowledge, no qualitative Canadian studies have been conducted regarding this topic. Fear surrounding childbirth must be fully understood from women’s perspectives so we can attempt to prevent experiences leading to childbirth fear and to inform the ongoing development and testing of comprehensive tools to measure fear and provide women-focused, trauma-informed interventions to deal with it. Prevention and early intervention have the potential to enhance women’s mental, physical, and psychosocial health. These may also reduce the number of maternal requests for cesareans due to fear and potentially lessen risks to mothers and newborns (SOGC, 2018).

## Purpose

We explored primiparous and multiparous women’s lived experiences of fear surrounding childbirth in relation to their decision to request a planned cesarean birth.

## Methods

### Study Design

We used Colaizzi’s (1978) phenomenological method to uncover the lived experience of fear surrounding childbirth from the women’s perspectives. Although his method is primarily descriptive, it also includes elements of interpretive phenomenology. Colaizzi did not believe that the researcher can completely “bracket” or disregard one’s own presuppositions about that experience, as would be expected in purely descriptive phenomenology. He suggested that researchers should begin by first uncovering and examining their presuppositions about the phenomenon to uncover their attitudes, beliefs, and hunches about the phenomenon. He recommended that the researcher use these presuppositions to help formulate research question(s) and then ask participants about their experiences to uncover additional information to construct a fuller meaning of the experience as described by those who have lived it.

### Sample

We sought a purposive sample and used snowball sampling as well. Participants were primiparas and multiparas who self-identified as having experienced childbirth fear that was instrumental in their request for or intent to request a planned cesarean. It did not matter how much time had elapsed since they gave birth. We reached data saturation with a sample of 16 women who lived in 4 Canadian provinces. They ranged in age from 18 to 50 years at the time of the interview (*M* = 37.5 years). Half of the women had never experienced perinatal loss, whereas the remainder lived through as many as five miscarriages, a stillbirth, and a neonatal death. Refer to Table 1 for a complete description of the participants’ characteristics.

**Table 1.** Participant Characteristics (N = 16).

Characteristic	n	%
Marital status		
Married/common law	13	81
Single/widowed/divorced	3	19
Parity		
Primipara	7	44
Multipara	9	56
Highest level of education		
High school/some college/university	5	31
College/university	5	31
Postgraduate degree	6	38
Family’s yearly income		
≤50,000	3	19
51,000–100,000	6	37
>100,000	7	44

### Recruitment and Data Collection

We used several methods to recruit women. Posters were placed in obstetricians’, public health nursing, and pediatricians’ offices; at family resource centers; on the postpartum and pediatric units and ultrasound departments of two general hospitals on Prince Edward Island; and at various locations at the University of Prince Edward Island. We also used a breastfeeding moms’ Face Book site. The first author was interviewed by local and national media and received numerous emails from women across Canada who met the inclusion criteria.

Data were collected through in-depth interviews. Most were conducted in person, although women from out of the province were interviewed via SKYPE or telephone. Women chose the setting for the in-person interviews. Most preferred their home; however, some chose the researcher’s private office. All participants consented to be audiotaped. The interviews lasted between 1/2 and 1 3/4 h. Women also shared blogs and journals. In keeping with Colaizzi’s (1978) method, all women were asked: “Can you please share your thoughts and feelings about the fear you experienced or are experiencing surrounding your childbirth? Please talk about your fear at any time. . .before, during pregnancy, labor and birth, postpartum and give examples.” Appropriate probes were used at the end of the interview to encourage women to clarify statements or provide additional examples.

Before initiating our study, we obtained ethics approval from the university and provincial research ethics boards. The first author described the study and obtained written consent from all women. They were made aware of the voluntary nature of the study and their right to withdraw at any time during and after the interview. Participants were asked for permission to use quotations and they chose pseudonyms to help protect their anonymity.

We enhanced the trustworthiness of the study using Lincoln and Guba’s (1985, 2000) five categories of credibility, dependability, confirmability, transferability, and authenticity. This was achieved by listening intensively during interviews, probing carefully to ensure rich and comprehensive data, audiotaping and transcribing interviews verbatim, ensuring prolonged engagement during data collection until saturation was reached, using reflexive journaling and examining/re-examining presuppositions throughout the study, keeping an audit trail, employing investigator triangulation, and ensuring researcher credibility. Additionally, we emailed the exhaustive description of the phenomenon to all participants as a form of member checking. The following is Elaine’s feedback:

It was lovely to see your name in my inbox, and even lovelier - if strange, like looking through time - to read through this thoughtful and sensitive draft. Your care with everyone’s narratives shines through here. . .The themes resonated for me and I have nothing particular to add. . .I just wanted to. . .let you know I was smiling at you as I read.

### Data Analysis

We also used Colaizzi’s (1978) method of data analysis. Colaizzi recommended seven steps to analyze the written descriptions, referred to as “protocols.” However, he acknowledged that there may be overlapping among them and “that the listed procedures and their sequences should be viewed flexibly and freely by each researcher” (p. 59) so they can be modified appropriately depending on the approach and phenomenon. In Table 2, we present the details of Colaizzi’s data analysis steps. From each participant’s description of the phenomenon, we extracted significant statements which were sentences or phrases that directly described the phenomenon. From each statement, we created formulated meanings that, according to Colaizzi, the researcher must cautiously take a leap from what the participant said to what she means staying true to what the participant said. This is where Colaizzi’s connection to interpretive phenomenology is enacted. In Table 3, we provide a partial audit trail of formulated meanings derived from women’s significant statements for Theme Three. We then organized all significant statements/formulated meanings into clusters of themes and integrated the results into an exhaustive description of the phenomenon. As noted, we emailed the exhaustive description to all women and, based on their feedback, no changes were made. Lastly, we formulated a statement of the fundamental structure of the phenomenon, a condensed statement of the exhaustive description.

**Table 2.** Colaizzi’s Data Analysis Protocol.

Data analysis steps
(a) Read all of the descriptions (protocols) to get a feeling for them and make sense of them; (b) Return to each protocol and extract significant statements or phrases that pertain directly to the phenomenon; (c) Formulate the meanings for each significant statement (but allow the data to speak for itself); (d) Repeat steps 1, 2, and 3 for each protocol and then organize the formulated meanings into clusters of themes referring to original protocols to validate them (Ask: Is anything missing or is there something that is not in the original descriptions?) noting any discrepancies among or between various clusters. Tolerate ambiguity at this point; (e) Integrate the results into an exhaustive description of the phenomenon; (f) Formulate the exhaustive description into a statement of its fundamental structure; (g) Return to participants for a validation of the findings and reformulate if necessary, based on participant feedback (Colaizzi, 1978).

**Table 3.** Examples of Formulated Meanings from Significant Statements for Theme 3.

Significant statement	Formulated meaning
“I, I guess, it felt overwhelming. And it at times, it stopped me in my tracks. (Pause). And overwhelming, feeling made me feel paralyzed.” (Andrea)	The fear felt overwhelming and stopped her in her tracks. The overwhelming feeling made her feel paralyzed.
“Like it’s just all-consuming and you can’t do anything but experience it. And you can’t get away from it, it’s just like a, I don’t know. It’s just intense.” (Sienna)	The fear is all consuming. She can’t get away from it and must just experience it and it is intense.

## Results

### Fundamental Structure of Phenomenon

During the analysis of the 16 interviews, we extracted 710 significant statements from which we generated 5 overarching themes, the exhaustive description, and the fundamental structure of the phenomenon *women’s experiences of fear surrounding childbirth*. The following is the fundamental structure of this phenomenon. Women described numerous antecedents of childbirth fear. Many revealed a previous experience in their lives that they perceived as physically or emotionally traumatic. Most described more than one fear surrounding childbirth. The vast majority expressed a fear of their baby/babies being injured or dying. Most were also fearful of a loss of control during the labor process. They also feared that they themselves might die or develop complications during childbirth. Many also expressed a fear of experiencing a physically or emotionally traumatic birth. Several women did not readily disclose their fears, as they believed that they would not be taken seriously or understood by others. Women used many vivid descriptions about living with fear. The vast majority described numerous emotional and physical manifestations of fear and depicted it with respect to time: time in their pregnancy and time of day. Most women experienced the fear constantly; however, it was worse at night. All women voiced that gaining control and planning for their birth helped them cope with their fear(s). They requested a planned cesarean birth because they believed this would provide more control over the birth process. Not all women eventually had a planned cesarean; however, they all considered it as a way of coping with their fear(s). Women talked about carefully weighing the pros and cons of cesarean versus vaginal birth to help them come to their decision; they did not make this decision lightly. Validation by others (professionals, family, and friends) and giving the women voice were very helpful in managing their fear(s). They described internal coping mechanisms such as visioning, mindfulness, and prayer. Women also explained about using self-protection: detaching from, not telling others about, delaying, or terminating the pregnancy. Others found that seeking knowledge, being distracted, finding a creative outlet, being reassured of fetal wellbeing, and developing a contingency plan helped them cope with their fear(s). Half of the women suggested that they had experienced some resolution or healing. For most of these, the birth of their healthy baby/babies began the healing process, whereas others noted that their fear resolved or subsided when it was confirmed that they would have a planned cesarean, that their doctor of choice would attend their birth, and that they had a choice in their birth plan which was validated by others.

With respect to the five overarching themes, themes one and two set the stage for why women requested a planned cesarean and included the antecedents of fear and the actual fears women experienced. The remaining themes depicted what it was like for women to live with these fears, how they coped with them, and whether they experienced any resolution of or healing from their fears.

### Theme 1- Setting the Stage: Antecedents of Fear

There were numerous antecedents or sources of the fears described by the women. Most multiparas reported that their fear arose from a previous birth experience during which they suffered physical or emotional trauma, in some cases leading to posttraumatic stress disorder. Physical trauma included requiring an emergency cesarean birth or an episiotomy and experiencing pain in labor; a medicalized birth; or birth complications such as an inverted uterus, shoulder dystocia, or postpartum infection. Emotional trauma arose from a dehumanizing birth, a loss of confidence in and separation from one’s body, fetal distress, a near-death experience, stillbirth and neonatal death, and severe infant morbidity. Elaine, whose first son died within hours of his birth, and with her second birth had an episiotomy done without her consent remarked:

I can honestly speak to the fact that with a traumatic birth, even when you bring home a healthy baby, it’s really fucking traumatic in its own way. We need to not completely shove that aside or excuse dehumanizing women in the birthing process simply for the sake of the baby. I had this particularly unique experience on both sides and both sucked.

Most women also attributed their fear to knowledge, either having too much or not enough knowledge about the birth process. Some talked about being fearful due to a lack of choice surrounding childbirth, hospital routines, and the feeling of being confined during labor. Several, noted that the fear arose from previous miscarriage(s) or because they were experiencing a high-risk pregnancy. Others, mainly primiparas, recounted an experience with infertility, a preexisting fear of death since childhood or a near-death experience, childhood sexual abuse, young age, lack of support, and a preexisting medical condition. Cloe, who had experienced infertility, miscarriages, and a twin pregnancy following in vitro fertilization remarked, “Like. . .so much of them being conceived was out of my control and being unable to conceive on my own that once I had them in me, I wanted to protect them and keep them safe.” Toni described, “So, um, my fear in terms of childbirth I guess stems back to um since I was a little girl. . . it stems back to my fear of death. . .”

Others expressed that their fear was a result of a lack of trust in health care providers (physicians and nurses). Lauren described her loss of trust in nurses following a dehumanizing experience during her first birth experience:

You know in the beginning I was trusting, but once I figured out that I couldn’t trust them anymore as to what they were doing. . . Like if I could have physically gotten up out of bed and marched down the hallway and got the doctor, I would have done that myself.

### Theme 2- So Many Fears: Not Always Easily Disclosed

Most women described more than one fear surrounding childbirth. The vast majority expressed a fear of their baby/babies being injured or dying during childbirth. Most participants feared a loss of control and a general fear of labor. Cloe described her primary fear:

. . .because I’m terrified that if we don’t control the birth, that if we go for a vaginal birth, something will go wrong. And my anxiety over vaginal birth and them [twins] being somehow injured or killed or something is creating a lot of anxiety for me. Like the whole natural birth of, it’s uncontrolled, everything’s out of our control.

Others expressed fear of the unknown and fear of medical and hospital routines, labor pain, hospitals in general, and epidurals. They also feared that they themselves might die or develop complications during childbirth. Many women were fearful of experiencing another traumatic birth, an episiotomy, tearing, poor/dehumanized care, a medicalized birth, and being violated or exposed. Women also expressed a fear of failure. Kara, who had experienced previous miscarriages, divulged her fear of failure:

So I think this whole piece of my womanhood that was consumed by pregnancy and childbirth and having children, um, to just have to accept that you might fail at that was really tough and created a level of fear about the whole process.

Other women were afraid of an emergency cesarean birth and had fears about physicians. They feared being cared for by a physician with who they previously had a bad experience, were fearful of their current physician, or were afraid they would not get their preferred doctor due to on-call systems. Those who experienced infertility were afraid they would never be parents. Lastly some participants expressed a fear that the birth would interfere with bonding and breastfeeding postbirth, they would have a lack of support from family, and their coworkers or partners would not be able to cope with their birth.

Although women discussed their fears with their physicians, some shared that they waited until later in pregnancy and others reported that they had never told their entire story to anyone before. They suggested that the medical community and society, in general, do not make it easy for women to talk about their experiences. They did not readily disclose their fears, as they believed that they would not be taken seriously or understood by others. Toni’s statement illustrates this sentiment:

. . . like if we can collectively as a society. . .learn to take women a little bit more seriously . . .really start to value their concerns and emotional wellbeing, I think that in the medical world . . . that it’s completely swept under the rug and it’s just such a crucial part of becoming a mother. Birth is remembered. It is a peak experience that only happens once or twice in most women’s lives and it affects them, for the rest of their lives. . . it is such a crucial part of a woman’s life. . .It’s not just healthy baby, healthy mom. . .that matters. The emotional wellbeing for that mom can be life or death for some.

### Theme 3- Living with Fear: The Looming Monster

The women used many vivid descriptions regarding what it was like for them to live with the fear(s) they experienced. The majority disclosed numerous emotional manifestations of fear using descriptors such as “terrified, horrified, petrified, irrational thoughts, overwhelmed, sense of doom, dark emotion, vulnerable, powerless, bundle of nerves, walking on eggshells, worst nightmare, flashbacks/crippling images, overpowering, torture, inability to concentrate, sadness, dark cloud, turmoil, lack of enjoyment, robbed of pregnancy, controlling, consuming, personality changes, uptight, and tense.” Lucy, who had experienced five miscarriages, exclaimed, “It was a monster. Oh yeah you knew it was there. . .I think there are things it robbed me of. . .just being present in that experience.” Sienna, who had experienced a previous stillbirth, described her response to fear as “a hamster on a wheel, mind spinning.” Elaine remembered that “it was very visceral.” May commented, “. . .just like racing thoughts, I couldn’t focus, especially at school. . .” Zoe remarked, “It is raw.” Darlene described fear as “the perfect storm.”

Most of the participants also recounted a variety of physical manifestations that arose when experiencing fear. Many described “trouble breathing and tightness in their chest/chest pain.” Elizabeth disclosed, “But yeah the breathing, you know, not being able to catch my breath and feeling overwhelmed. . .” Samantha remembered, “They were tightening in my chest type thing, like where you feel like someone is sitting on you, couldn’t get a full breath. . .” Others experienced “shaking, a fight or flight reaction, tingling in hands, panic, faint, urinary frequency, and tightness in shoulders/tense.” Heidi remembered, “But I would feel that kind of trapped sense of panic about 2 or 3 months in every time that. . .I have to do this.. . .And I mean you are. . .kind of trapped in your body.” Grace remarked, “Its um, I can even feel it a little bit now while I talk about it, a little bit of the sweaty, flushed face.” Toni described her physical manifestation of fear during her second labor when she realized that the physician who inverted her uterus by pulling on the cord with her first birth was in the labor room across from her:

I literally shut down. . .went blank. . .I said, ‘I’m not moving. I don’t want to be here. I want to go home. . .I can’t do this’. . . I just shut down and I didn’t talk to anybody, I just stayed in like a shell of that fear. . . and I just stayed there. . .wouldn’t look at anybody. . .I was paralyzed.

Kara summed up the all-encompassing nature of fear. She explained, “Well it affects you on so many levels. It affects you mentally, physically, spiritually, it affects every aspect of your life.”

Most women also described fear with respect to time: time in their pregnancy, time of day, constant, or recurrent. The majority experienced the fear constantly; however, it was worse at night. A number depicted the fear as pervasive or intrusive and some experienced horrifying or vivid nightmares. Cloe remarked, “It’s just so pervasive. . .the fear went everywhere with me. I wasn’t without it for a second of the pregnancy. . .Every minute of that 9 months, I was afraid to lose those babies.” Lucy remembered, “it loomed here all the time. . .Constant. It never, I don’t think it ever left me.” Andrea shared, “Yes, it’s going to be 9 months of hell and it was, it was horrible.” Cloe recounted a vivid, recurring dream:

The other dream that I had I was going for the section and. . .you know being awake and waiting for the doctor to deliver the babies and feeling the mood in the room change, because the doctor and the nurses knew that the babies were dead when you know. . .I’ve never seen a section video or anything, but in my dream it was just like, opening me up, and in my dream I could see these two. . .lifeless, blue-faced babies.

### Theme 4- Coping Strategies: Taking Control of the Uncontrolled

All women voiced that gaining control and developing a plan for their birth helped them cope with their fear(s). Requesting a planned cesarean birth was the way they all believed that they would have more control over the birth process. Not all women eventually had a planned cesarean; however, they all considered it as a way of coping with their fear(s). Lauren could not bring herself to become pregnant for several years after her first traumatic birth experience and knew from the minute she got pregnant she was having a planned cesarean. She stated, “Our second child. . .was born 7 and 1/2 years after our first. . .I had discussions with my obstetrician. . .and I told him the very first day that I went in that I wanted to have a planned C-section.” Other women planned a change in hospital or had a mental plan in place.

Several women revealed feeling a “tug of war” carefully weighing the “pros and cons” of cesarean versus vaginal birth to help them make their decision. Zoe’s comment exemplifies this. “I know there’s other issues that can happen. You can have bad infection. . .issues with your uterus. . .I know there’s a lot of risk to it, but I know that it was the right thing I had one.”

Validation by professionals, family, and friends and giving the women voice was very helpful for them to cope with their fear(s). Heidi remarked, “when they [doctors and nurses] actually listened to my concerns. . .as if they were valid. . .and explained what was going on . . .then I felt less fear and more calm. . .” Lauren’s comment exemplifies “giving voice”:

We own our bodies and we deserve respect and ability to make decisions about how our bodies are treated and I think a lot of times in the medical field that’s kind of forgotten, that your patients have a voice. And I certainly made sure that my voice was well heard the second time around.

Many participants talked about internal coping mechanisms that helped them deal with their fear(s). These included “visioning, mindfulness, self-talk, magical thinking, prayer, compartmentalizing, and systematic thinking.” Elaine, who felt violated during her second birth due to lack of consent for an episiotomy, described one of her coping methods. “I had done an extraordinary amount of like mental unpacking work just to kind of just get that image of the scissors out of my mind.” Some women used self-protection as a coping mechanism such as detaching from the pregnancy, not telling others about the pregnancy, delaying pregnancy, or terminating the pregnancy. Darlene stated:

You were just this bundle of nerves. Like we didn’t take a picture of me pregnant until we were ready to go in for a section, because I was convinced that something traumatic was, if it didn’t happen during the pregnancy, there would be something traumatic happen when I had the baby.

After initially considering a planned cesarean for her third pregnancy, Florence had an abortion. She remarked, “. . ..I chose to terminate because there was no way on God’s earth I was going through that [traumatic episiotomy] again.”

Other women found that seeking knowledge, using distraction, finding a creative outlet (blogging, art, journaling), being reassured of fetal wellbeing (using a home doppler, having ultrasounds, visiting the ER, and poking the baby), and developing a contingency plan helped them cope with their fear(s). May sought information about cesarean births before making her decision. She stated, “So I actually did all the research. . . I watched videos of both. . .” Chloe described her efforts to manage her fears:

Like I said, the night before the section, I had these nightmares. And you know I was waking up in sweats. You know I couldn’t even get to sleep. . . I’d poke here, I know baby B was here and she’d kick me back. And I’d poke here, and baby A would kick me back. And I was like okay, they’re both still alive. We did the doppler on both spots, found two different heartbeats. . .

Elaine blogged, “I feel silly about my fear. . .but it is real and grows bigger as I do. Birth has marked me in ways I do not like. I do not want to dwell on those wounds or give them power.”

### Theme 5- Resolution: Beginning the Healing Process

Half of the women experienced some resolution or healing from their experience. For most of them, the birth of their healthy baby/babies began the healing process, as well others felt healing begin when they could breastfeed their newborn(s). Andrea, whose first son was severely disabled from childbirth, remembered, “Uh the fear lasted right up until I heard the baby cry, it lasted right up until the very moment.” Zoe, who was fearful that one or both of her twins would have cerebral palsy, remarked, “. . .the release of it [fear] was wonderful after just nursing the twins.” Others noted that their fear resolved or subsided when it was confirmed that they would have a planned cesarean, their doctor of choice would attend their birth, and they had a choice in their birth plan that was validated by others. May shared, “I felt 100% better from him saying, like ‘okay I’m going actually going to book the date’.” Elaine recalled the promise her obstetrician made to her, “. . .the biggest thing that she did was that she promised me that if I went into labor. . .that she would come in, that I could call her, and she would come in. . .which meant the world to me.” Chloe disclosed, “I think the fear was extinguished over time. . .”

## Discussion

All of the women in our study experienced fear surrounding childbirth, some more severe than others, and each requested or intended to request a cesarean birth because of that fear. Our findings add to our understanding of women’s lived experience of fear surrounding childbirth and suggest that there are many similarities between Canadian women and those from other countries. The first two overarching themes and theme clusters reaffirm those reported in the literature (Dencker et al., 2019; Eide et al., 2019; Karlström, 2017; Melender, 2002; Poikkeus et al., 2016; Ryding et al., 2016); however, the rich descriptions provide a deeper and more comprehensive understanding of the antecedents of fear and the actual fears women experienced.

Most multiparous women recounted that their fear arose from a previous physically or emotionally traumatizing birth. Primiparous women tended to describe other sources of trauma such as childhood sexual abuse, frightening information from others, miscarriage, and infertility. Dencker et al. (2019) also reported that the sources of fear between multiparous and multiparous women are not always the same. One similarity between these antecedents of fear is that most women suffered a previous physical or emotional trauma that led to their fear(s). This may or may not be a birth trauma. This is an important finding as it highlights the importance of trauma-informed care.

Most of the fears that women identified in our study also have been reported in the literature (Eide et al., 2019; Long et al., 2018; Nilsson & Lundgren, 2009; Poggi et al., 2018; Sheen & Slade, 2018; Slade et al., 2019). Importantly, several of the women in our study were not able to readily disclose these fears. They believed that their fear(s) would not be understood by others and society in general. Similar findings have been reported by several authors (Eriksson et al., 2006; Nilsson & Lundgren, 2009; Wigert et al., 2020). Although not necessarily a new finding, it underscores the importance of listening to women and validating their fears.

Our participants recounted very vivid and sometimes horrific descriptions of living with their fear(s). Many became tearful during the interview when describing their experiences, sometimes even after many years after giving birth. Nilsson et al. (2012) reported that every childbirth becomes “etched” in women’s memory and these memories are very detailed, emotional, and vivid even after many years. It was evident that these women suffered emotionally and physically, most throughout their entire pregnancy and some before and after. Experiencing physical manifestations (Eriksson et al., 2006), nightmares (Saisto et al., 2001), anxiety (Nerum et al., 2006), interference with occupational and academic functioning (Wijma, 2003), feeling “captured” by the pregnancy and by the fact that they could not turn back (Nilsson & Lundgren, 2009; Wigert et al., 2020), and feeling a loss of control (Nilsson et al., 2010) have been previously reported. However, many of the manifestations have not been previously described and expand our understanding of what women may experience and why we need to encourage them to express their lived experience.

Women recounted the numerous ways they coped with their fears and gained control of or power over them. This expands our understanding of the many ways women cope with fear. Requesting a planned cesarean birth to control the uncontrollable labor experience was the primary way women found to cope with their fears. The decision to request a cesarean was not made lightly. Many women struggled with this decision, weighing the “pros and cons” in a “tug of war.” They knew there were risks to having a cesarean birth but, for the most part, they believed that controlling the birth was a safer option and outweighed the risks to them and their baby/babies. Most women in Eriksson et al.’s (2006) study also described weighing the pros and cons of cesarean birth; however, women in our study were more explicit about this *tug of war* and reminds us as providers to understand the dilemma they face. Validation of their fears and birth choice by others (professionals, family, and friends) and giving women voice was an important coping mechanism. Eriksson et al. (2006) reported similar findings. Even though not all women had a cesarean, knowing that it was an option if needed assisted their coping. They felt a sense of being listened to and validated. This finding underscores the approach recommended by the SOGC (2018) and suggests that if there is open communication, women feel they have been a partner in decision making, and trust their provider, they may be able to give birth vaginally. Having a physician of their choice was also important to several women in our study. This reflects one deficit of the on-call system that women find themselves facing. Lastly, several of the participants in our study expressed a sense of healing. For some, the healing process began after the birth of their healthy baby/babies, whereas for others it began upon confirmation that they would have a planned cesarean or have their doctor of choice. Confirmation of a planned cesarean to assist the resolution of fear is an important learning from our study.

Numerous implications for practice arise from our findings. Being aware of the antecedents of women’s fears is important. We must focus on prevention of these fears when possible and must have a comprehensive understanding of the sources to do this. Preventing physical and emotional birth trauma is critical and often this is under the control of caregivers. Humanizing the birth process is essential. Behruzi et al. (2014) purport that humanizing birth requires personalization, human caring, recognition of the woman’s rights, advocacy, and companionship “and a balance between medical care and comfort, safety, and humanity” (p. 127). Providing information to and communicating with pregnant women in a sensitive, trusting, caring manner, and giving women choice and control in their birth plan are also essential. Assessing women for risk factors that might lead to fear and assessing their level of fear and coping mechanisms using a comprehensive, woman-centered, trauma-informed approach must become a routine component of perinatal care. As providers, we need to give women the voice to express their fears and the agency to help them cope with them. Addressing and validating their fears by being gentle, empathetic, kind, respectful, and supportive; offering existing community resources; and developing evidence-based interventions to address their specific fears is essential. Additionally, health professionals must remember the significance of women’s birth experience and not focus solely on safety surrounding birth. Lastly, as a society, we need to give women voice to talk about their fears and to validate their experiences.

Further qualitative research is required to develop a deeper understanding of the fears surrounding childbirth and coping strategies of specific populations of women such as those who have suffered perinatal loss, infertility, high-risk pregnancy, and sexual abuse. This will assist practitioners to address their unique needs. We must continue to develop and test tools that assess a broad range of fears and their antecedents. This will help increase the consistency across studies when measuring the concept of fear surrounding childbirth. It also will increase the comprehensiveness and quality of tools for use in clinical practice to assess women’s fears. Further high-quality randomized controlled trials are required to develop and test evidence-based interventions that can be used by practitioners to assist women to deal with their fears surrounding childbirth.

The trustworthiness of our findings may have been reduced by the cultural homogeneity of our sample. It also may have been decreased by potential differences in data quality between in-person, SKYPE, and telephone interviews.

In conclusion, our findings provide a deeper understanding of fear surrounding childbirth from Canadian women’s perspectives, particularly those who request a cesarean birth due to fear. Prevention of, assessment for, and early intervention for fear(s) have the potential to enhance women’s physical, mental, and psychosocial health and to help validate or legitimize their experiences. We must advocate for women, give them voice, validate their fears, and end their suffering in silence.
